# Highly Sensitive Active-Matrix Driven Self-Capacitive Fingerprint Sensor based on Oxide Thin Film Transistor

**DOI:** 10.1038/s41598-019-40005-x

**Published:** 2019-03-01

**Authors:** Guk-Jin Jeon, Seung-Hwan Lee, Seung Hee Lee, Jun-Bo Shim, Jong-Hyun Ra, Kyoung Woo Park, Hye-In Yeom, Yunyong Nam, Oh-Kyong Kwon, Sang-Hee Ko Park

**Affiliations:** 1 Korea Advanced Institute of Science and Technology, Department of Materials Science and Engineering, 291 Daehak-ro, Yuseong-gu, Daejeon, 34141 Republic of Korea; 2 Hanyang University, Department of Electronic Engineering, 222, Wangsimni-ro, Seongdong-gu, Seoul, 04763 Republic of Korea

## Abstract

The fingerprint recognition has been widely used for biometrics in mobile devices. Existing fingerprint sensors have already been commercialized in the field of mobile devices using primarily Si-based technologies. Recently, mutual-capacitive fingerprint sensors have been developed to lower production costs and expand the range of application using thin-film technologies. However, since the mutual-capacitive method detects the change of mutual capacitance, it has high ratio of parasitic capacitance to ridge-to-valley capacitance, resulting in low sensitivity, compared to the self-capacitive method. In order to demonstrate the self-capacitive fingerprint sensor, a switching device such as a transistor should be integrated in each pixel, which reduces a complexity of electrode configuration and sensing circuits. The oxide thin-film transistor (TFT) can be a good candidate as a switching device for the self-capacitive fingerprint sensor. In this work, we report a systematic approach for self-capacitive fingerprint sensor integrating Al-InSnZnO TFTs with field-effect mobility higher than 30 cm^2^/Vs, which enable isolation between pixels, by employing industry-friendly process methods. The fingerprint sensors are designed to reduce parasitic resistance and capacitance in terms of the entire system. The excellent uniformity and low leakage current (<10^−12^) of the oxide TFTs allow successful capture of a fingerprint image.

## Introduction

As various mobile devices have become widely used, desired information is accessible easily anytime and anywhere. The growth of mobile devices has led to the development of various industries related to these devices, and such accompanied growth has turned the information technology platform into a new paradigm of the internet of things (IoT). Biometric identification technologies also have been developed in line with the increasing importance of personal security in the IoT era^1,2^. Recent biometric identification devices utilize mainly unique and behavioral characteristics, such as face, iris, vein, fingerprint, and so on. The fingerprint has been the most widely used feature for biometric identification because fingerprint identification technology has many advantages, such as a very reliable identification technique, portability, easy-to-use method, high recognition rate, and low maintenance cost^3^.

The detection of fingerprint can be realized by optical^4^, electrostatic^5^, ultrasonic^6,7^, mechanical, or thermal methods. The capacitive method has been commonly used in mobile devices. There are two types of capacitive fingerprint sensors (FPSs), mutual-capacitive and self-capacitive types^8^. Considering the FPS as a miniature touchscreen, the mutual-capacitive method can be easily applied to a FPS. The sensing method commonly uses a structure with an insulator between two electrodes, one for driving and the other for sensing. Contact of the fingerprint results in a reduction of mutual capacitance between the two electrodes, and thus there is a difference in capacitance between ridges and valleys. Multi-touch functionality in the mutual-capacitive method^9–11^ enables the realization of FPS, whose multiple pixels are touched simultaneously by a fingerprint^5,12^. The ridge-to-valley capacitance (C_ridge-valley_) obtained from a mutual-capacitive FPS, however, is even lower than parasitic capacitance (C_p_), resulting in low sensitivity^13,14^. The mutual-capacitive FPS with most commonly used diamond-shaped patterns has been reported^12,15^. Since the maximum C_ridge-valley_ was 5fF and C_p_ was 10 pF due to the sensor structure when 1-μm-thick SiO_2_ was used as the overlaid layer, C_p_ was 2000 times higher than C_ridge-valley_. Such a large difference required a complicated sensing circuit in order to recognize a fingerprint, which could increase production costs. In the self-capacitive sensing method, a unit cell is conventionally composed of a sensing plate and a readout circuit based on Si-based technologies^16–18^. Since it is individually addressed by a controller, and the self-capacitance of a sensing electrode induced by a fingerprint is sensed directly and independently, the method can have low ratio of C_p_ to C_ridge-valley_, resulting in high sensitivity^10^. However, since Si-based FPSs are fabricated only on opaque, brittle and rigid Si wafer, its applications are limited to hard devices such as the home button of a smartphone. Without addressing the sensor by ICs connected individually to each pixel, the self-capacitive sensing method is also disadvantageous to achieve multi-touch functionality due to the ghost issue^19^. To combine the aforementioned advantages of each method, a matrix-type self-capacitive FPS with switching devices, which electrically isolate the pixels in the FPS, should be developed. Thin film transistors (TFTs) based on low-temperature polycrystalline silicon semiconductor, amorphous silicon semiconductor, or oxide semiconductor can be applied to the FPS for the promising performance of the sensor, large area process compatibility, and application even to a display front panel. In particular, the oxide TFT is considered a good candidate for addressing the self-capacitive FPS due to its moderate mobility, high on/off ratio, low process cost, and applicability to even transparent and/or flexible substrates^20–24^. To increase the security level of the self-capacitive FPS even in a small size, high resolution is also important while maintaining high sensitivity^25,26^. Accordingly, the oxide TFTs are strongly needed to secure high resolution and sensitivity of a FPS.

Herein, we demonstrate a self-capacitive FPS with a resolution of 500 pixels per inch (ppi) and an area of 1 cm^2^ by employing a high mobility Al-InSnZnO TFT. According to the Federal Bureau of Investigation (FBI), FPSs used in FBI should have higher resolution than 250 ppi^27^. The resolution of our FPS are two times higher than the criteria of FBI. Since the area of our FPS are wider than that of conventional FPSs, which mostly have an area below 1 cm^2^, our FPS can include more information of a fingerprint. We adopted a back-channel etch (BCE) structured TFT fabricated by the same techniques as used in the display industry. Based on simulations of the FPS anticipating C_ridge-valley_ in the array, the materials and devices are designed and fabricated to successfully extract a fingerprint image from our FPS.

## Results

### Simulation and experiment of capacitance change in sensing electrode

**Figure 1:**
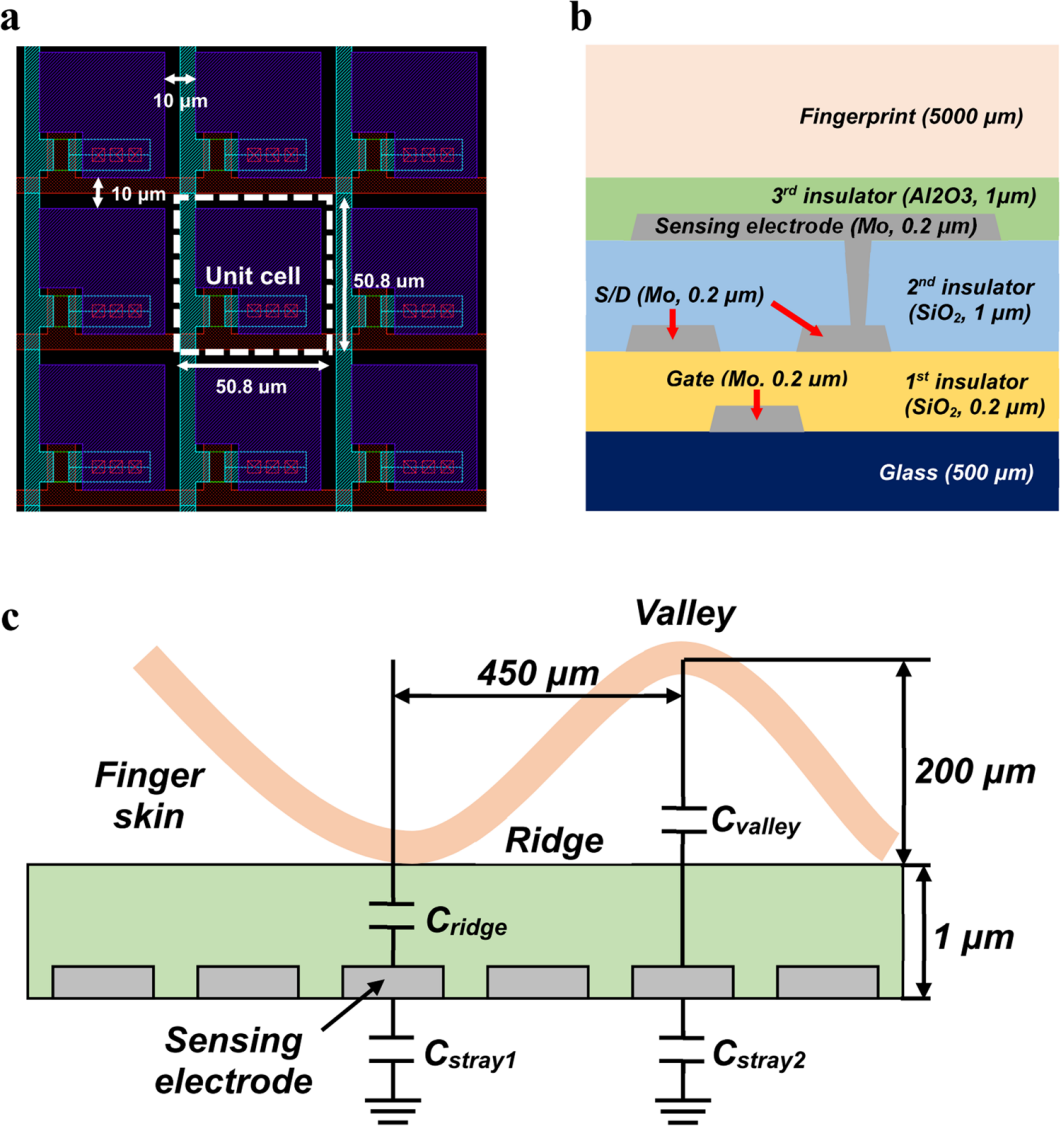
Design and simulation to confirm the feasibility of fingerprint sensor. (**a**) Design scheme of unit cell of fingerprint sensor with a resolution of 500 ppi. (**b**) Cross-sectional device structure of fingerprint sensor with the assumption that the sensor has a back-channel etched oxide TFT. (**c**) Cross-sectional structure for simulation of capacitance difference between a ridge and a valley.

**Figure 2:**
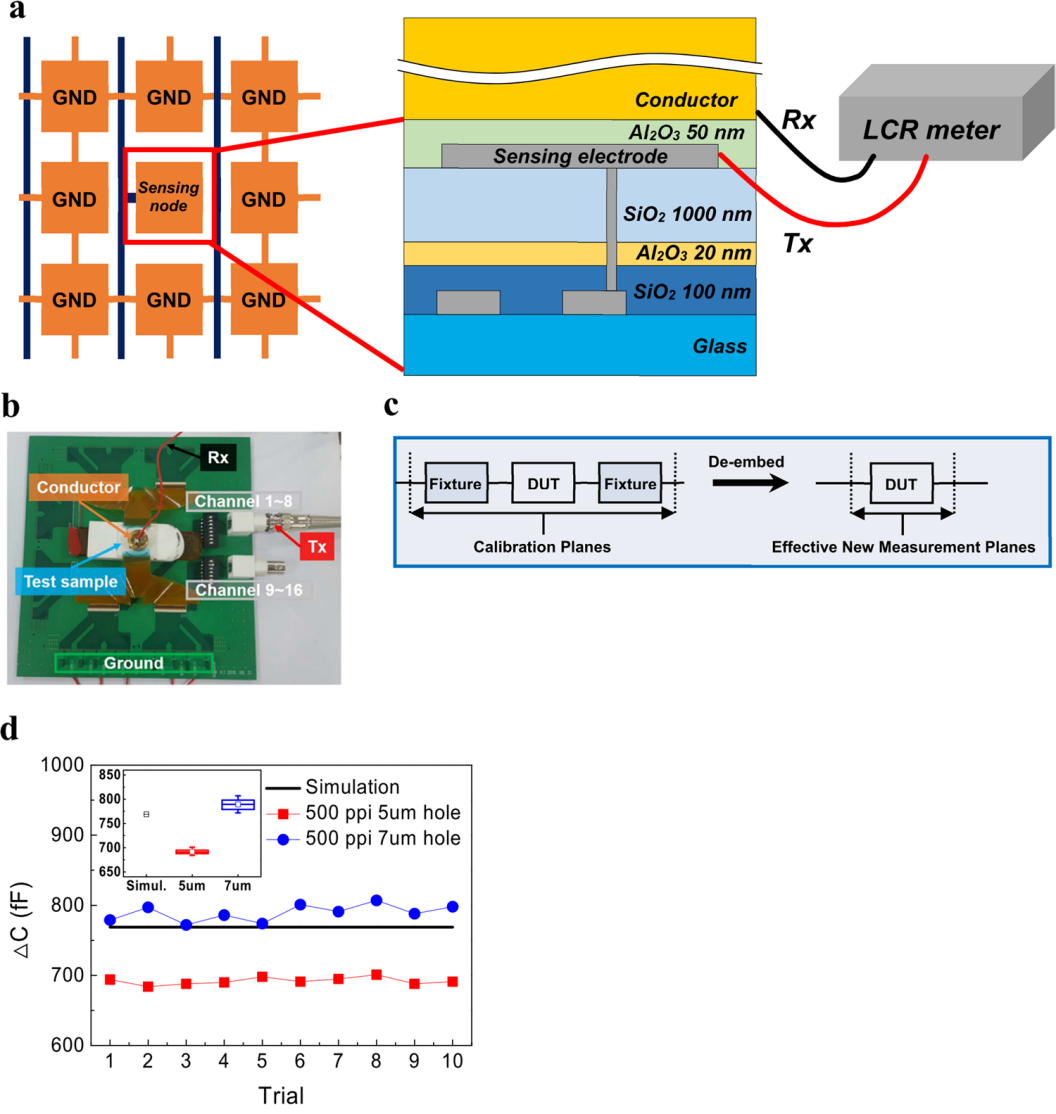
Measurement method and results to confirm capacitance change from specific sensing nodes. (**a**) Schematic structure of system for measuring capacitance of specific nodes. (**b**) Real image of capacitance measurement system. (**c**) De-embedding method for accurately measuring the capacitance of a sensing node. (**d**) Comparison of simulation and experiment on the capacitance change of specific sensing electrodes in the sensors without a TFT in the pixel with a resolution of 500 ppi according to 5 μm and 7 μm metal contact holes. The inset shows the error bars of capacitance change.

We targeted a FPS with a resolution of 500 ppi. The main sensing area was designed to be 1 cm × 1 cm among the total sample area of 2.5 cm × 2.5 cm. First, a unit pixel was designed in the layout of array (Fig. 1a). The longitudinal and lateral length of a unit pixel were 50.8 μm, where the longitudinal line and lateral line were the data line and driving line, respectively. The sensing part was formed in the shape of a square with an empty part. The pitch of sensing electrodes was determined to be 10 μm to mitigate parasitic capacitance. Each layer of the TFT, sensing electrode, and insulator between the sensing electrode and finger was designed with materials that can be applied to a large area process such as Mo, SiO_2_, and Al_2_O_3_ (Fig. 1b). The capacitance difference between a ridge and a valley is dependent on the capacitance of an insulator above a sensing electrode. We applied a 1-μm-thick Al_2_O_3_ film with a dielectric constant (ε_r_) of 7.6 to the overlaid layer for the simulation (Supplementary Figs S1 and S2). As illustrated in Fig. 1c, we assumed that a ridge and a valley of a fingerprint touched the surface of the overlaid layer well. The spacing between the ridge and the valley and the depth of the valley were modeled to be 450 μm and 200 μm, respectively, which are within the typical fingerprint size range^28^. Each capacitance was represented by C_ridge_, C_valley_, and C_stray_, where C_ridge_ and C_valley_ indicate the capacitance from the Al_2_O_3_ film to a ridge and a valley, respectively, and C_stray_ expresses a fixed electrostatic capacitance that exists between the sensing electrode and the ground. We also considered the capacitance per unit cell caused by the overlap between the gate and drain electrodes (O_gate-drain_) and the overlap between the scan and data lines (O_scan-data_), C_line_. The simulated values are listed in Supplementary Table S1, where the self-capacitance of the sensing electrode is expressed by C_self_. Since the width/length of the O_gate-drain_ and the O_scan-data_ were 2 μm/13 μm and 5 μm/5 μm, respectively, the C_line_ was about 8.12 fF by Raphael simulation and the C_stray_ was about 20fF. The parasitic capacitance, which is the sum of C_line_ of 196 pixels, and C_stray_, was calculated to be about 1.61 pF while scanning a pixel. Note that the capacitance difference between C_self(ridge)_ and C_self(valley)_ was about 108 fF. The C_p_ of 1.61 pF was about 15 times higher than the capacitance difference of 108 fF. Since custom-designed capacitance sensing ICs, which were used mainly for touch screens, were used to detect C_self(ridge)_-C_self(valley)_, we evaluated whether the simulated C_self(ridge)_-C_self(valley)_ could be distinguished from the C_p_. In the case of our existing 5-inch and 32-inch touch screens, the parasitic capacitance was about 40 times and 450 times higher than the capacitance changed by a finger, respectively. Therefore, we anticipated that the sensing ICs can distinguish ridges from valleys. Even if we used 1-μm-thick SiO_2_ as the overlaid layer instead of 1-μm-thick Al_2_O_3,_ the ratio of C_p_ to C_self(ridge)-Cself(valley)_ in our FPS was about 31 considering ε_r,_ and still much lower than that of mutual-capacitive FPS aforementioned. Next, we investigated experimentally how large the self-capacitance of the sensing electrode is. First, our sensors were fabricated to connect a specific sensing electrode with a specific data line without a TFT in the pixel. The sensing electrodes surrounding the specific sensing electrode were grounded so as not to influence the measurement (Fig. 2a). Since it would be very difficult to touch a ridge or a valley of a fingerprint on a certain sensing electrode, we placed a flat conductor on the total surface of the sensor. Figure 2b shows the real measurement system used to measure the capacitance of a specific sensing electrode while placing the conductor on the surface of the sensor. Sixteen sensing electrodes were connected with the transmission probe of a LCR meter through switches to individually select each electrode. The conductor was connected with the receiving probe of the LCR meter. As shown in Fig. 2c, a de-embedding method was used to remove the influences of parasitic capacitance in the existing fixture and correctly obtain the experimental values^29,30^. The capacitance of a sensing node was obtained by subtracting the remaining capacitance except for the capacitance of a specific sensing electrode from the total capacitance. The sensing electrodes of 10 points were randomly selected and measured. The measured values are similar to the simulated capacitance of 769 fF (Fig. 2d). Because of our sensor structure, the capacitance could be dependent on the metal contact hole size, which connects the source electrode and the sensing electrode, and the area of the sensing electrode. In the sensors with a resolution of 300 ppi and 500 ppi, holes with a size of 5 μm or 7 μm were applied to our devices. As the metal contact hole size of the sensing electrode increased, the capacitance was similar to the simulated value (Fig. 2d and Supplementary Fig. S3). In order to investigate the influence of the hole size, the current versus voltage characteristics of a pattern with 100 holes connected together by a metal electrode were measured (Supplementary Fig. S4). The resistance obtained from the pattern with the hole size of 7 μm was lower than that of the pattern with the hole size of 5 μm. The increase of capacitance related to the hole size is attributed to the reduction of contact resistance through the holes. The lowest percentage error between the experimental and simulated values was 2.64% in the device with a hole size of 7 μm and a resolution of 500 ppi. Because the experimental value was in fairly close agreement with the simulated value, we determined that the capacitance change of the sensing electrode could be detected by our system.

### Fabrication of FPS integrating Al-doped InSnZnO TFT

**Figure 3:**
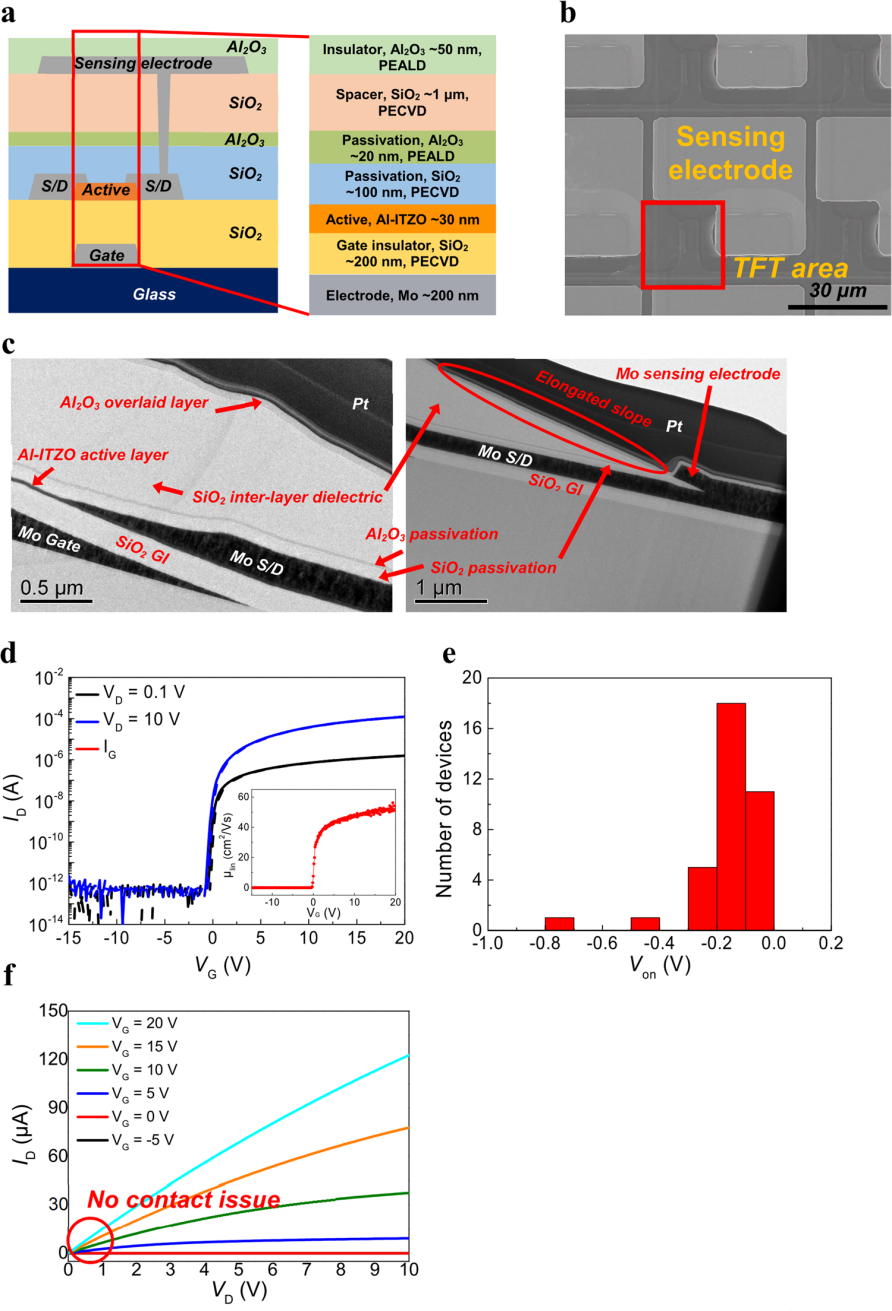
Structure of fingerprint sensor and performance of oxide TFTs. (**a**) Cross-sectional structure of fingerprint sensor in a pixel. (**b**) SEM image of fingerprint sensor. (**c**) TEM images of cross-sectional structure of fingerprint sensor. (**d**) Transfer characteristics (V_D_ = 0.1 and 10 V) of Al-ITZO TFT. The inset displays the linear mobility as a function of V_G_. (**e**) Distribution histogram with turn-on voltage of 36 of TFT devices on a 10 cm × 10 cm glass substrate. (**f**) Output characteristics (V_G_ = −5, 0, 5, 10, 15 and 20 V) of Al-ITZO TFT.

A FPS integrating Al-doped InSnZnO (Al-ITZO) TFTs with field-effect mobility higher than 30 cm^2^/Vs was fabricated by the processes shown in Fig. 3a. A Mo film with a thickness of 200 nm was deposited by DC sputtering and patterned as a gate electrode on glass substrates. After the formation of the gate electrode, SiO_2_ film with a thickness of 200 nm was deposited as a gate insulator (GI) by PECVD at 380 °C, followed by the deposition of an Al-ITZO active layer of 30 nm under oxygen partial pressure of 30%. A Mo film with a thickness of 200 nm was also deposited and patterned for a source/drain electrode. Oxygen was supplied into the back channel of the TFTs by N_2_O plasma treatment in a PECVD chamber to reduce oxygen vacancies of the back channel^31^. Next, a SiO_2_ passivation layer with a thickness of 100 nm was deposited *in situ* with N_2_O and SiH_4_ gases at 300 °C. An Al_2_O_3_ film with a thickness of 20 nm was deposited on the SiO_2_ passivation layer at 200 °C by plasma-based ALD to passivate the active layer completely. The channel width and length of all TFT devices are 5 μm and 5 μm, respectively. A SiO_2_ inter-layer dielectric with a thickness of 1 μm was then deposited by PECVD at 300 °C in order to reduce parasitic capacitance below the sensing electrode. For metal contact with a sensing electrode, a SiO_2_/Al_2_O_3_/SiO_2_ layer was patterned and wet-etched at once. A Mo film (200 nm) was deposited and patterned for a sensing electrode, followed by deposition of a 50 nm Al_2_O_3_ film for an overlaid layer. The Al_2_O_3_ overlaid layer was patterned and opened to form a metal contact pad for bonding with the flexible printed circuit board (FPCB). Lastly, the FPSs were post-annealed at 300 °C in a vacuum to reduce some shallow defect states and improve the electrical performance of the TFT^32–34^. Figure 3b shows a top image of the FPS from a scanning electron microscope (SEM). The Al-ITZO TFT was designed to be overlapped as little as possible the sensing electrode in a pixel so as to reduce parasitic capacitance. In our FPS array, the sensing time can be affected by a combination of parasitic resistance and capacitance, which is called RC delay^35^. Therefore, the parasitic components were considered in order to confirm the feasibility of adequate operation speed in our fingerprint sensing system. The remaining parasitic capacitances result from a metal line from the sensor to the pad, the flexible printed circuit (FPC) cable, and the printed circuit board (PCB) (Supplementary Table S2). The parasitic resistance is composed of the metal line in the sensor, the metal line from the sensor to the pad, the metal line in the FPC cable, and the metal line on the PCB. Among them, the parasitic resistance based on the metal lines in the sensor and from the sensor to the pad is the dominant factor. The total parasitic resistance and capacitance are expected to be 6.323 kΩ and 4.485 pF, respectively, and the RC time constant, which indicates the delay of a signal, is about 28.35 ns. Therefore, the cutoff frequency, which is a boundary in the frequency response of our system, is determined to be about 5.61 MHz. This cutoff frequency was higher than 2 MHz, the operating frequency of our sensing IC for the detection of capacitance change. This indicates that our FPS can operate at a sufficient driving speed by our circuit.

The surface of the FPS was investigated by a surface profiler. The sensing electrodes were scanned from right to left (Supplementary Fig. S5a). Most region of the sensing electrode was observed to be flat, which is beneficial for conformal contact of the fingerprint, while some of the sensing area protruded about 200 nm because of the source/drain data line extending up and down (Supplementary Fig. S5b). Since the protruding shape is negligible compared to the size of a human fingerprint, we anticipated that the shape would not present a problem in detecting the fingerprint. In Fig. 3c, the transmission electron microscope analysis showed that our FPS had a clear interface between each layer, and all layers were accurately deposited to the desired thickness.

The performance of the Al-ITZO TFTs was electrically characterized. The transfer characteristics of an Al-ITZO TFT are displayed on a semi-logarithmic scale in Fig. 3d. The Al-ITZO TFT has a subthreshold swing (SS), hysteresis, and turn-on voltage (V_on_) of 130 mV/dec, 1.82 V, and −0.44 V, respectively. The inset in Fig. 3d shows a plot of the linear mobility (μ_lin_) versus the gate voltage (V_G_ at V_D_ = 0.1 V). The μ_lin_ value is extracted to be 50.18 cm^2^/Vs at V_G_ = V_on_ + 15 V. All TFT devices had a high on/off ratio of 10^7^.

The V_on_ of the TFTs distributed in an area of 10 cm × 10 cm was also investigated. All of the TFT devices were completely depleted in a V_G_ of −1 V (Fig. 3e and Supplementary Fig. S6a). The SS, hysteresis, and μ_lin_ values of the TFT devices are presented statistically in Supplementary Fig. S6b–d. Even though all parameters show slight differences, this does not present a significant problem in operating the FPS. Because the off-current of the TFT devices increases with the number of pixels that are connected with a data line, low off-current of a TFT is very important to detect a small difference by a fingerprint during the turn-on of a pixel. The TFTs have off-current less than 1 pA, which is low enough compared to the on-current of the TFT. Figure 3f shows the drain current versus source-to-drain voltage (I_D_ − V_D_) output characteristics of this Al-ITZO TFT with various values of V_G_ in 5 V steps. The output curve of the TFT exhibits linearity of I_D_ in the low V_D_ region and saturation of I_D_ in the high V_D_ region. Since I_D_ is modulated linearly depending on the change of V_G_ and V_D_ in the low V_D_ region of the output curve, it is determined that the contact issue is not observed.

As is well known in the display industry, the oxide TFTs generally have critical instability against prolonged bias stress, temperature, and light illumination, resulting in a threshold voltage shift due to the charge trapping in the GI or at the GI/channel interface, defect generation in the channel, and abnormal phenomena such as a transfer curve with a hump^34,36–38^. Since the Al-ITZO TFTs integrated in our FPS are subjected to row-by-row scanning at V_G_ between −10 V and 20 V periodically, it is necessary to confirm the electrical stability performance of the oxide TFTs. Therefore, we conducted various stability tests under positive bias temperature stress (PBTS), negative bias temperature stress (NBTS), and negative bias illumination stress (NBIS). Supplementary Fig. 6a,b show the PBTS and NBTS results under V_G_ = ± 20 V and V_D_ = 0.1 V at a temperature of 60 °C. The V_on_ shifts under PBTS and NBTS were 2.24 V and −1.68 V, respectively. Positive V_on_ shifts were observed in the PBTS test with no degradation of SS and μ_lin_, which indicated that the V_on_ shifts resulted from charge trapping without defect creation. In the NBTS test, V_on_ was shifted positively after stress for 30 s by filling the shallow defect states located near the interface between the GI and the active layer, which resulted in hysteresis of TFTs. The remaining NBTS results were shifted in parallel to the negative direction after stress for 10 ks. Many FPSs have been frequently applied to mobile display devices such as smartphones and table PCs. The FPSs can be spontaneously exposed to light illumination in mobile display devices. Therefore, the electrical stability of oxide TFTs based on light illumination was investigated by a NBIS test. The Al-ITZO TFT was stressed electrically and optically under V_G_ = −20 V and V_D_ = 0.1 V at light intensity with a power density of 0.5 mW/cm^2^ (Supplementary Fig. S7c). The V_on_ was shifted negatively by about −4.9 V with degradation of the SS, which indicated defect creation. The negative V_on_ shift of the oxide TFT under NBIS has been explained with the direct injection of holes generated by light illumination into the valence band of the gate insulator, and/or trapping into the bulk trap of the gate insulator by the ionized oxygen vacancies generated by the capture of holes^36^. The oxide TFT in our FPS showed acceptable stabilities under the various stress tests.

### Extraction of fingerprint image from FPS

**Figure 4:**
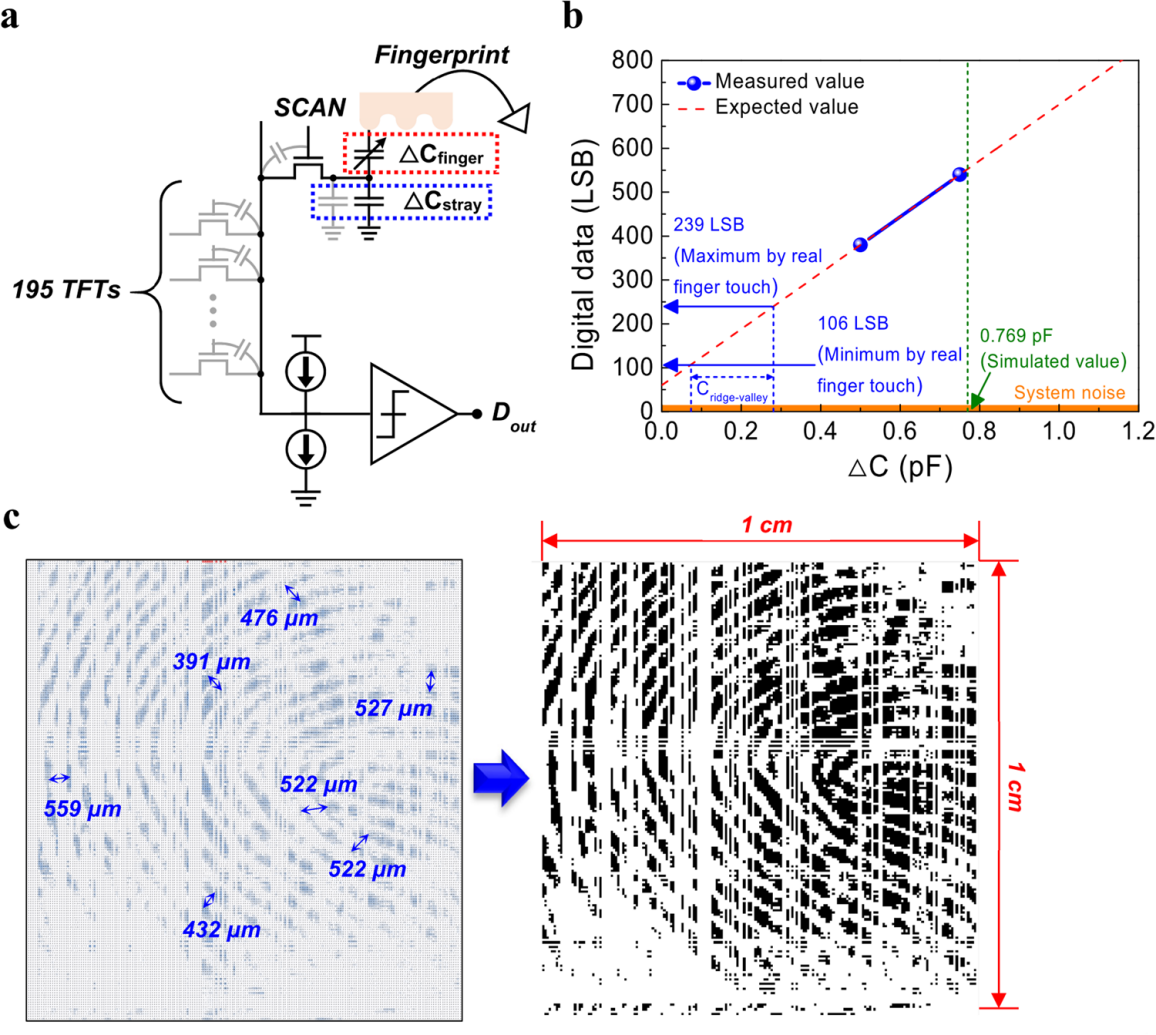
Sensing mechanism and performance of fingerprint sensor. (**a**) Equivalent circuit of fingerprint sensor. (**b**) Change of digital data depending on capacitance change. The blue circles were the values obtained by connecting ceramic capacitors of 0.5 and 0.75 pF with a sensing line on the PCB of fingerprint sensor. Since we used the sensing ICs whose the output digital values were linearly changed depending on the input capacitance, the red dashed line was obtained by linearly fitting the blue circles. The previously simulated value on the capacitance change of a sensing electrode and the averaged maximum and minimum digital values, which were acquired by real finger touch, were displayed by the arrows. The C_ridge-valley_ indicates the capacitance difference between a ridge and a valley. (**c**) Raw data (left) and processed image (right) acquired from fingerprint sensor with a resolution of 500 ppi.

The sensing mechanism of our FPS is expressed by the equivalent circuit in Fig. 4a. When a scan line is operated, a data line is repeatedly charged or discharged. If a ridge or a valley touches the surface of the FPS, the capacitance of the sensing electrode increases or remains unchanged, respectively. The increase of capacitance results in a delay of the charging or discharging signal in the data line due to RC delay. Finally, we can acquire the output digital data depending on the degree of variation from the initial signal. The circuit system is composed of a field programmable gate array (FPGA), a shift register/level shifter that produces voltage for on/off of switching TFTs, a sensing IC, and FPS boards (Supplementary Fig. S8). The control signals transferred from the timing controller of the FPGA are applied to the 196 scan lines of the FPS after passing through the shift register/level shifter of the main board. By the control signal applied to the scan line, the sensing node is selected row-by-row, and the readout circuit senses the self-capacitance value of the sensing node selected through the data line on the upper and lower sides. The acquired digital data are transferred to the receiver of the FPGA and stored in one frame unit. The stored digital data are transferred to the PC, and the fingerprint image is outputted through the image viewer in the PC. The digital data of system was monitored by connecting the ceramic capacitor elements with a sensing line on the PCB of FPS in order to expect the digital data obtained from the capacitance change of a sensing electrode when touching a finger. When the ceramic capacitor elements of 0.5 pF and 0.75 pF were connected with the sensing line, the digital values were about 380 lowest significant bit (LSB) and 540 LSB, respectively (Supplementary Fig. S9). The digital values measured by the ceramic capacitors were shown in Fig. 4b. Since our sensing IC linearly changed the output digital values depending on the input capacitance, the expected values were expressed by linearly fitting two values. Considering the expected curve and previously simulated value of 0.769 pF, we expected that the digital data would be about 560 LSB. When touching real finger on the FPS with a resolution of 500 ppi, the corresponding digital values were 239 LSB and 106 LSB, which were obtained from the average of maximum and minimum values depending on each channel respectively. We found out that the real capacitance difference between a ridge and a valley, C_ridge-valley_, was about 207 fF. Since the difference of digital values by C_ridge-valley_ was higher than the system noise, we determined that the fingerprint could be detected by our FPS system. The capture of fingerprint image was performed using our FPS system. We obtained the output digital values from the system with and without a finger touching the FPS. The final digital values were derived from the difference between two values. We considered higher values as ridges on the basis of average, threshold, depending on each channel. Figure 4c shows the single image of a fingerprint captured in 2 Hz frame rate using our FPS system. In consideration of the ratio of working pixels to total pixels in the digital data, the pixels corresponding to 68.8% worked normally. When we calculated the pitch between adjacent ridges from the fingerprint image, it ranged from 400 μm to 600 μm and was people’s general pitch range^28^. Therefore, we determined that the FPS system successfully captured the fingerprint image.

## Discussion

In summary, highly sensitive self-capacitive FPS based on Al-ITZO TFTs was demonstrated. Primarily, the state of fingerprint touching the surface of FPS was modeled with appropriate capacitive components and simulation in order to investigate the values. We confirmed that the capacitance difference between a ridge and a valley can be distinguished from the parasitic capacitance by our sensing IC through the simulation. It indicates our FPS was well designed for the fingerprint recognition. The simulation and device fabrication for a systematic approach to imitate reality allowed us to predict the change of real capacitance on the sensing electrode. The increase in metal contact hole size increased the capacitance value to a level similar to the simulated value. When measuring the resistance of the patterns connected with a metal electrode through 100 holes, the resistance of the pattern with 7 μm holes was reduced by about 11% compared with that of the pattern with 5 μm hole. Accordingly, the increase in capacitance was likely to result from the decrease in resistance through the hole. Since the Al-ITZO TFTs have uniform properties and low leakage current, the TFTs could be integrated in each pixels of FPS. The FPSs were designed to mitigate parasitic resistance and capacitance in consideration of parasitic components such as the metal line resistance, overlap capacitance, and so on. Therefore, the fingerprint sensing system operated with sufficient operating frequency. The C_ridge-valley_ of 207 fF was obtained by the real fingerprint when the thickness of Al_2_O_3_ overlaid layer was 50 nm. Even if we used 1-μm-thick SiO_2_ as the overlaid layer instead of 50-nm-thick Al_2_O_3,_ the ratio of C_p_ to C_ridge-valley_ in our FPS was about 327 considering ε_r_ and thickness_,_ and still much lower than that of the state of the art for mutual-capacitive FPS. It indicated that our FPS had high sensitivity compared to mutual-capacitive FPS. However, the C_ridge-valley_ in our FPS can be improved by flattening the surface of FPS with the protruded shape in order to remove the air between a ridge and the surface of FPS. The excellent uniformity and low leakage current of oxide TFTs also enabled successful capture of a fingerprint image. Although we captured the fingerprint image, the quality of image was low due to the use of sensing ICs mainly for touch sensor, the reduced FPCB bonding yield, and the difficulty in the particle control. The high quality image can be obtained if sensing ICs for fingerprint sensor are developed and FPCB bonding yield is improved. The reliability issue of the FPS also remains a challenge due to the sensitivity of the oxide TFT to environmental factors and the thinness of the overlaid layer, which cause degradation of the device by a penetration of oxygen and moisture, and surface damage by the mechanical contact. Applying a hard coating film^39^ and a thick overlaid layer to the top of the FPS can be a future work for reliable fingerprint image capture by the FPS. As aforementioned, since the Si-based FPS are fabricated on Si wafer and has also high thermal budget, it is not applicable to flexible plastic substrates, which limits the application range of FPS. Therefore, we believe the proposed FPS is a remarkable new application of oxide TFTs and a first step for a flexible FPS based on oxide TFTs. In the future, our FPS can be fabricated on a polyimide-coated glass substrate while maintaining the fabrication conditions, and the FPS on a polyimide can be easily detached by laser lift-off process due to photon energy absorption at interface between a polyimide and a glass. In conclusion, all the methods allow us to realize a flexible FPS. In order to drive oxide TFTs, the maximum supply voltage of FPS was from 20 V to −10 V. The supply voltage range needs to be reduced for mobile devices. Since our oxide TFTs have high on/off ratio even in the lower voltage range, it indicates we can lower the operating voltage. Reducing a channel length of TFT and increasing a mobility of active layer can be also helpful to lower the operating voltage. The fingerprint recognition system were fabricated as a prototype to confirm the feasibility of the self-capacitive FPS based on oxide TFT. Accordingly, the sensing circuits had bulky components and used FPGA, which made the size of our circuits large compared to the FPS. The size of sensing circuits can be miniaturized by developing application specific integrated circuit (ASIC) for our FPS.

## Methods

### Fabrication of FPS without oxide TFTs in pixels

The FPSs were fabricated in the structure without oxide TFTs in pixels to experimentally confirm the self-capacitance of the sensing electrode. A Mo film with a thickness of 200 nm, which has a sheet resistance of 2 Ω/sq, was deposited and patterned to form data lines on a glass substrate by sputtering, followed by the sequential deposition of 100-nm-thick-SiO_2_ film, 20-nm-thick Al_2_O_3_, and 1-μm-thick SiO_2_ film. The metal contact holes at data lines connected with specific sensing electrodes to be measured were formed by photolithography and wet-etching. A 200-nm-thick Mo film was deposited and patterned for the formation of the sensing electrode. An Al_2_O_3_ film with a thickness of 50 nm was finally deposited for passivation of the device by plasma-enhanced ALD. The pads were opened as a connection between each pixel and an external circuit for the measurement.

### Fabrication of FPS integrating oxide TFTs in pixels

A Mo film was deposited by sputtering and patterned to form a gate electrode by photolithography. A SiO_2_ gate insulator was deposited on the gate electrode by PECVD and then a Mo film was formed as a source/drain electrode, followed by the deposition and patterning of the Al-ITZO active layer. The devices were passivated by a SiO_2_ film using PECVD and an Al_2_O_3_ film by PEALD. A 1-μm-thick SiO_2_ film was deposited directly on the passivation layer and metal contact holes were patterned and wet-etched for the connection between the source electrode and the sensing electrode. A Mo film was deposited and defined for the formation of the sensing electrode. Lastly, an Al_2_O_3_ overlaid layer was deposited and the pads to be measured were opened by a wet-etching process.

### Measurements

The capacitance of the sensing electrode was characterized by a LCR meter (Hewlett Packard 4284a precision). The transfer and output characteristics of the Al-ITZO TFTs were electrically measured by a semiconductor analyzer (Hewlett Packard 4156a). The superficial and cross-sectional morphologies were imaged by field emission scanning electron microscopy (FE-SEM, Hitachi, model S-4800) and transmission electron microscopy (TEM, FEI, model Tecnai G² F30 S-Twin), respectively. The surface of FPS was investigated by a surface profiler (Veeco, model Dektak-8).

## Supplementary information

Dataset 1, Dataset 2, Dataset 3, Dataset 4, Dataset 5, Dataset 6, Dataset 7, Dataset 8, Dataset 9, Dataset 10, Dataset 11

## Data Availability

All data generated during and/or analyzed during this study are included in this published article (and its Supplementary Information Files).
