# Supplementary material for: Highly Sensitive Active-Matrix Driven Self-Capacitive Fingerprint Sensor based on Oxide Thin Film Transistor
Source: Sci Rep. 2019 Mar 1;9:3216. doi: 10.1038/s41598-019-40005-x (PMC6397235; doi:10.1038/s41598-019-40005-x)
Supplement: Supplementary file 1 — Dataset 1, Dataset 2, Dataset 3, Dataset 4, Dataset 5, Dataset 6, Dataset 7, Dataset 8, Dataset 9, Dataset 10, Dataset 11 [file 41598_2019_40005_MOESM1_ESM.pdf]

Highly Sensitive Active-Matrix Driven Self-Capacitive Fingerprint Sensor based on Oxide Thin Film Transistor

Guk-Jin Jeon[1,+], Seung-Hwan Lee[2,+], Seung Hee Lee[1], Jun-Bo Shim[2], Jong-Hyun Ra[2], Kyoung Woo Park[1], Hye-In Yeom[1], Yunyong Nam[1], Oh-Kyong Kwon[2,*] and Sang-Hee Ko Park[1,*]

[1]Korea Advanced Institute of Science and Technology, Department of Materials Science and Engineering, 291 Daehak-ro, Yuseong-hu, Daejeon 34141, Republic of Korea

[2]Hanyang University, Department of Electronic Engineering, 222, Wangsimni-ro, Seongdong-gu, Seoul, 04763, Republic of Korea

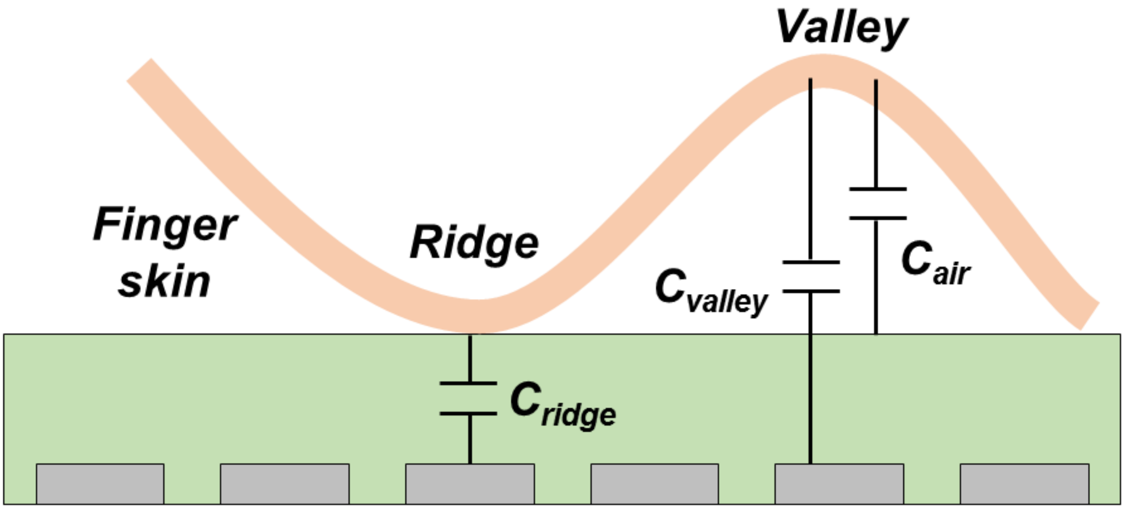

$$C_{valley} = \frac{C_{ridge}C_{air}}{C_{ridge} + C_{air}}$$

$$\Delta C = C_{ridge} - C_{valley} = \frac{C_{ridge}^2}{C_{ridge} + C_{air}} = \frac{C_{ridge}}{1 + \frac{C_{air}}{C_{ridge}}}$$

$$\varepsilon_r \; of \; overlaid \; layer \uparrow \rightarrow \; C_{ridge} \uparrow \rightarrow \Delta C \uparrow$$

Figure S1. Theoretical calculations about increase of capacitance difference between a ridge and a valley depending on the dielectric constant of the overlaid layer. As the dielectric constant of the overlaid layer is increased, the capacitance difference between a ridge and a valley is also increased.

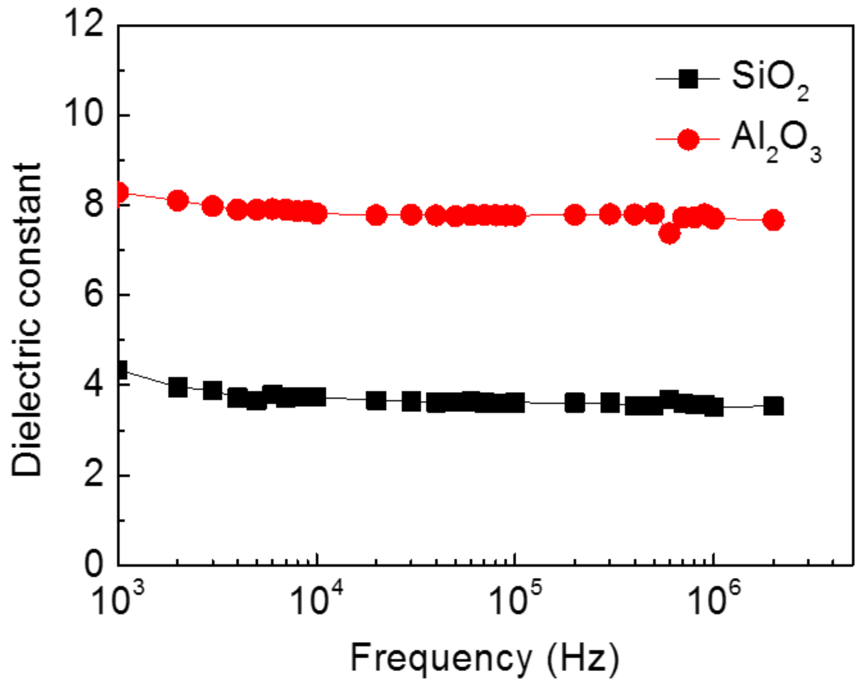

Figure S2. Relative dielectric constant (ε_r) extracted from the metal-insulator-metal (MIM) devices of 100 μm x 100 μm effective area with a 200-nm-thick SiO_2 and a 120-nm-thick Al_2O_3 dielectric layer. After the capacitance (C) values of MIM devices were measured depending on the frequency, ε_r values were calculated from the capacitance values using $\varepsilon_r = Cd/A$, where d is the distance between a top and a bottom metal, and A is the effective area.

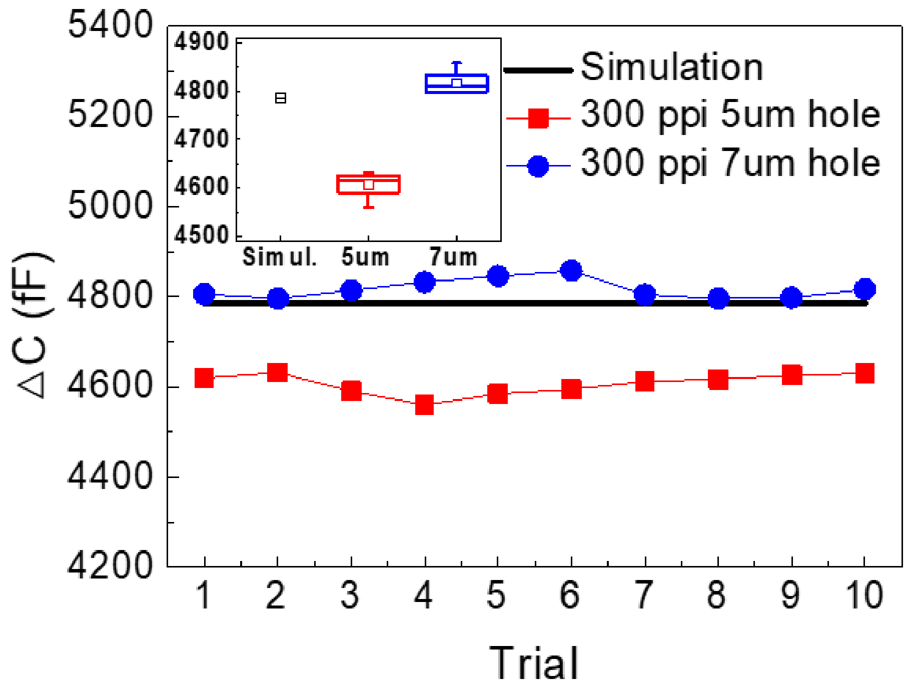

Figure S3. Comparison of simulation and experiment on the capacitance change of specific sensing electrodes in the sensors without a TFT in the pixel with a resolution of 300 ppi according to 5 μm and 7 μm metal contact holes. In the case of 300 ppi, the capacitance change of a specific sensing electrode in the sensor without a TFT in the pixel was also simulated and 4786 fF. The simulated value and the experimental values were compared, as shown in above figure. The increase in hole size affected the capacitance change of sensing electrodes to be similar to the simulated value. The inset shows the error bars of capacitance change.

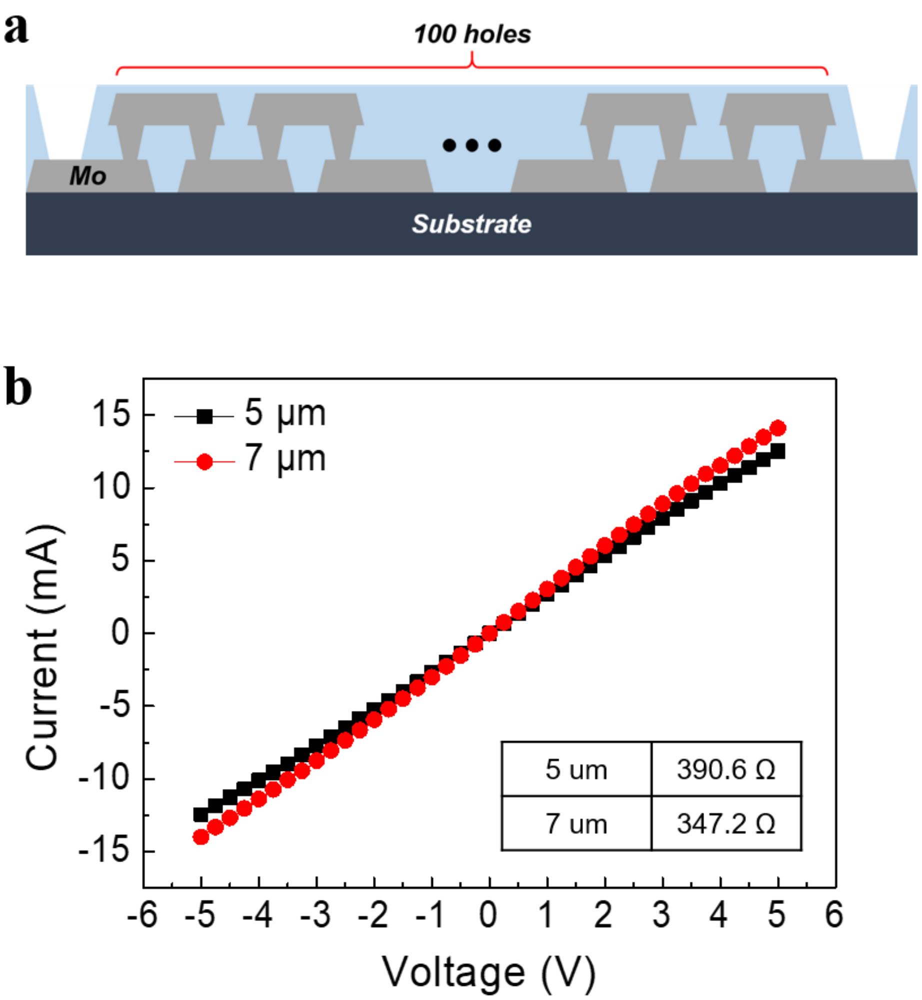

Figure S4. Electrical analysis of effect on the resistance through the holes depending on the metal contact hole size. (a) Structure of patterns with 100 holes with the size of 5 μm or 7 μm. (b) Current-voltage (I-V) characteristics of patterns with 5 μm or 7 μm holes. The resistance values were extracted from the slope of the I-V characteristics. Since the top and the bottom electrodes were connected only through 100 holes, we could investigate the influence of hole size.

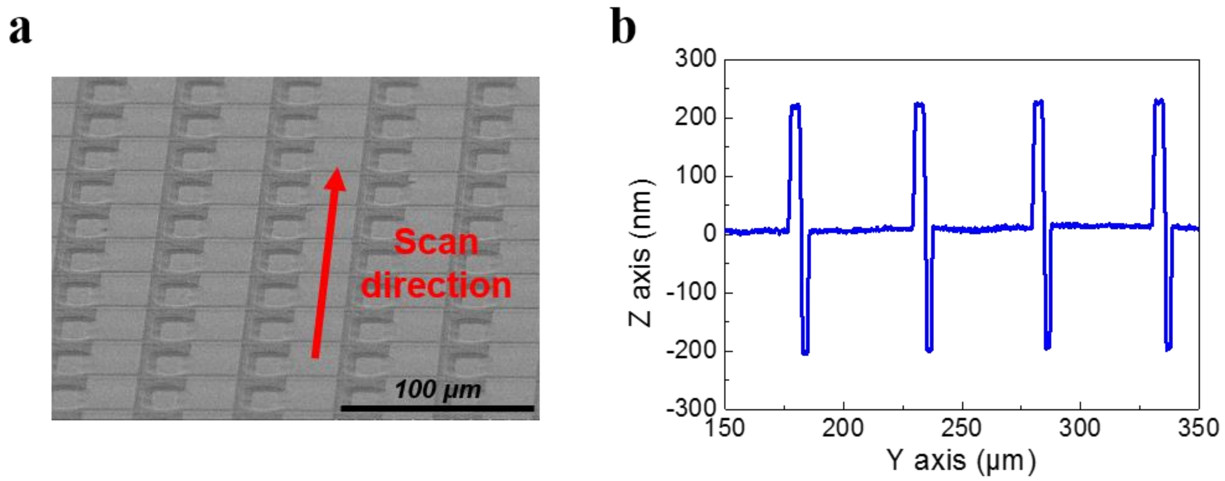

Figure S5. Surface profiler analysis of a top surface of fingerprint sensor. (**a**) Scan direction of surface profiler in a SEM image. (**b**) Scanning result of a top surface of fingerprint sensor. The SEM image shows the fingerprint sensor turned 90° to the right. As you can see in the SEM image, the probe of surface profiler moved from bottom to up on the surface of the fingerprint sensor.

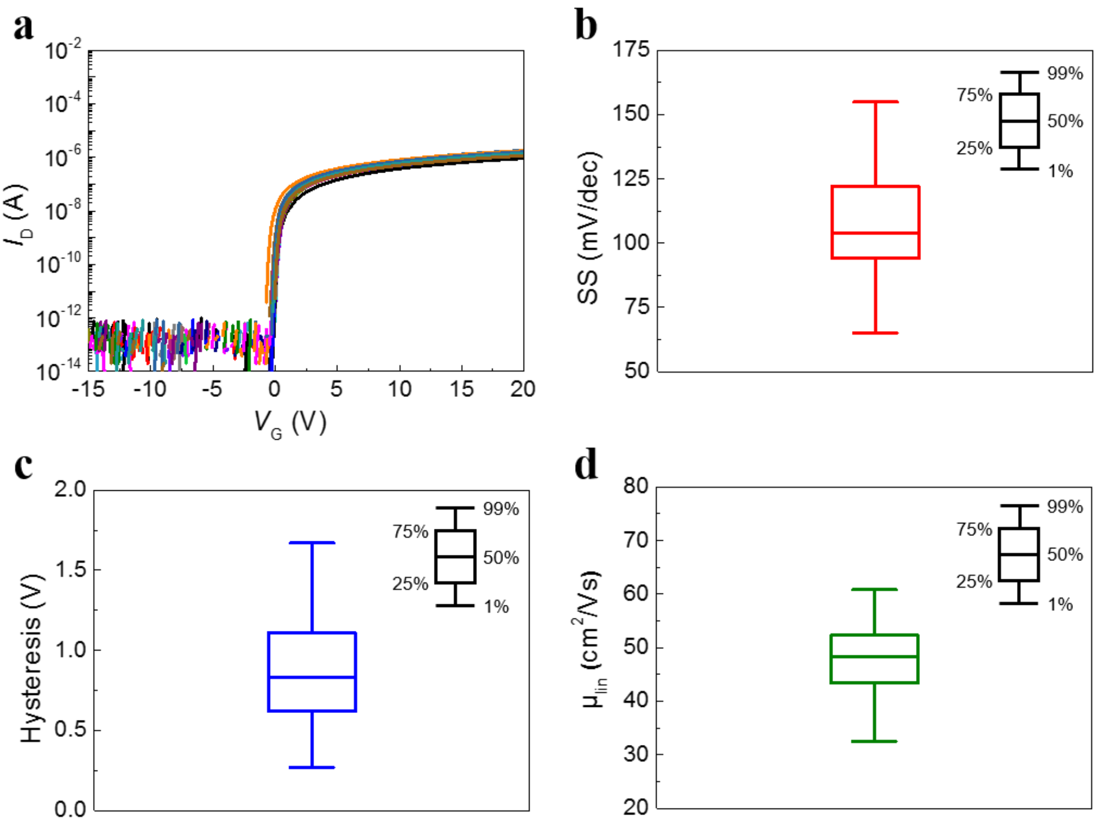

Figure S6. Statistical distribution of parameters obtained for Al-ITZO TFT devices. (**a**) transfer curves, (**b**) SS, (**c**) hysteresis, and (**d**) linear mobility of 36 of TFT devices randomly measured on a 10 cm x 10 cm glass substrate (V_D = 0.1 V). All parameters were extracted from the transfer curves of 36 of TFT devices. The insets in figure S6b-d indicate the percentile of parameters.

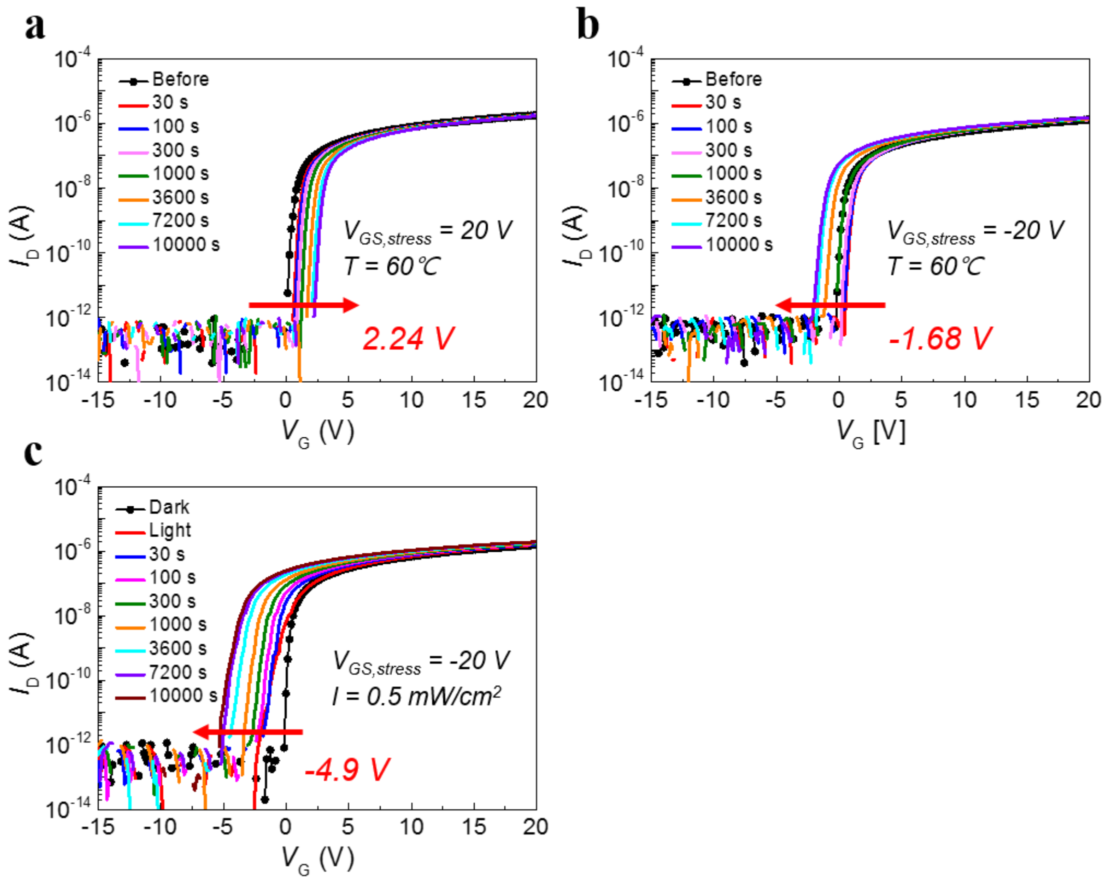

Figure S7. Stability performances of Al-ITZO TFT devices under (**a**) PBTS, (**b**) NBTS and (**c**) NBIS conditions for 10 ks. The bias applied on the gate of TFT ($V_{GS,stress}$), the temperature (T), and the illumination intensity (I) were 20 V, 60℃, and 0.5 mW/cm^2, respectively.

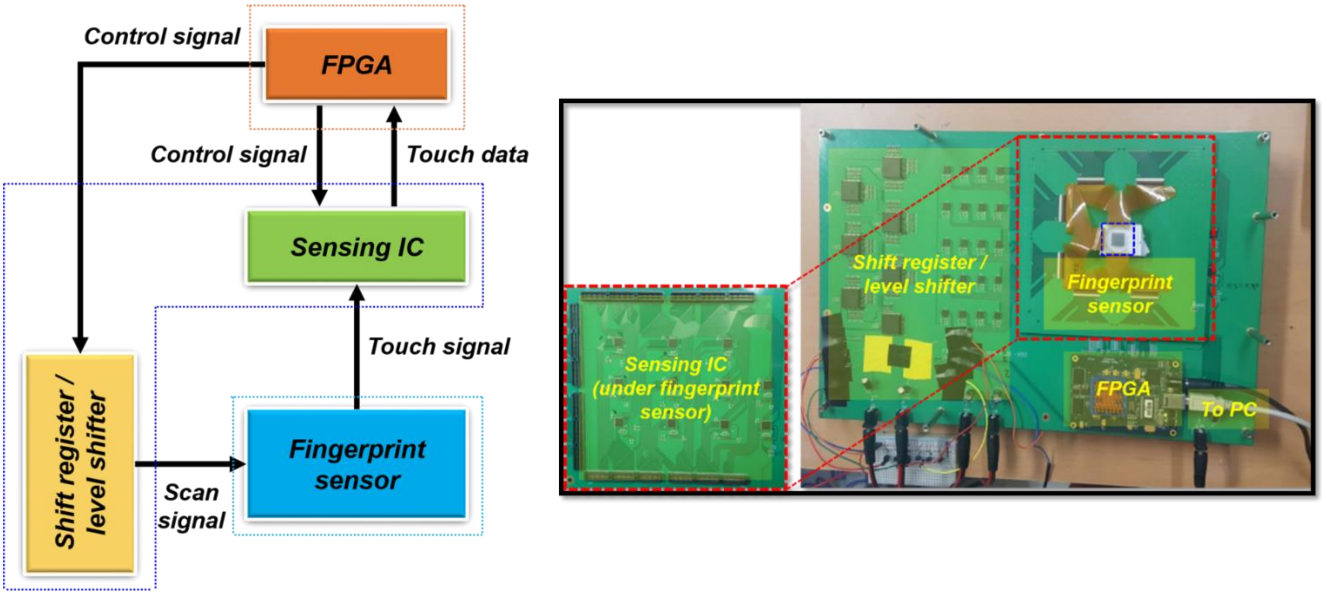

Figure S8. Schematic configuration (left) and real image (right) of fingerprint sensing system. The left figure briefly shows all systems shown in the right figure. In the right figure, since the sensing IC board was covered by the fingerprint sensor board, the sensing IC board was displayed separately.

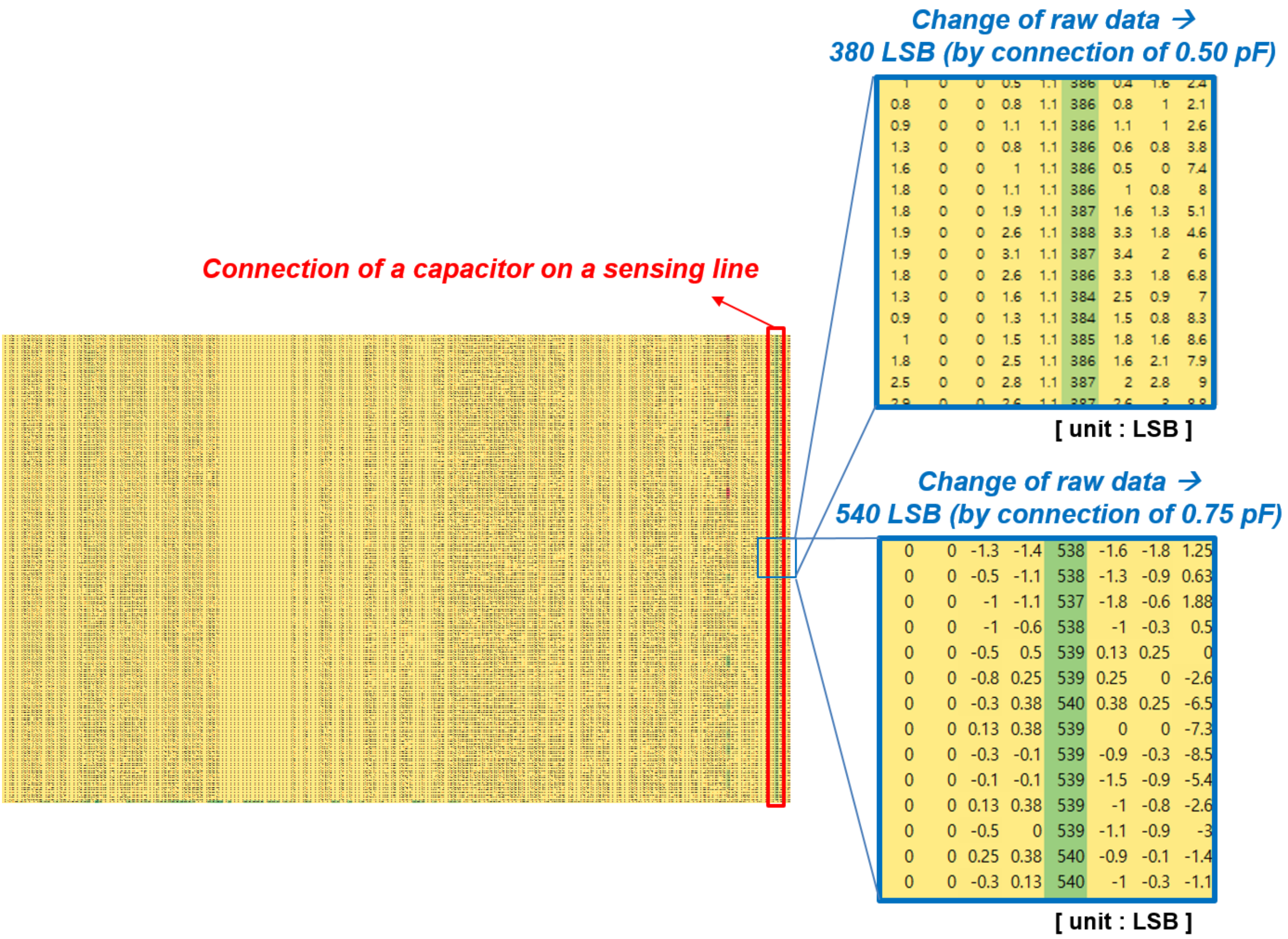

Figure S9. Change of digital data according to ceramic capacitors of 0.5 pF and 0.75 pF on a sensing line on the PCB of the fingerprint sensor. Assuming that the ridge of fingerprint was closely attached on the sensing electrode of the fingerprint sensor, the capacitance change of the sensing electrode was 0.769 pF calculated by the simulation. The ceramic capacitors with capacitance values similar to the simulated value were used in order to expect the digital data obtained by the capacitance change of sensing electrode when touching a finger on the surface of fingerprint sensor.

Capacitance	Value [fF]
$C_{self,untouch}$	20.1
$C_{self\ (ridge)}$	128.3
C_{ridge}	114.2
$C_{self\ (valley)}$	20.08
C_{valley}	0.09
**$C_{self(ridge)}$-$C_{self(valley)}$**	**108.2**
C_{stray}	20.1
C_{line}	8.12

Table S1. Simulated values of various capacitance, where C_{ridge} and C_{valley} mean the capacitance from Al_2O_3 film to a ridge and a valley, respectively. C_{self} means the self-capacitance change of a sensing electrode when touching finger on the surface of fingerprint sensor. C_{stray} represents a fixed electrostatic capacitance that exists between the sensing electrode and the ground. C_{line} indicates the capacitance per unit cell generated by the overlap between a gate electrode and a drain electrode and the overlap between a scan line and a data line due to the sensor structure.

Object		Value	Unit
On fingerprint sensor	Metal width	5	μm
	Metal length	10000	μm
	Line R	4	kΩ
	Unit C per cell	8.12	fF
	Line C	1.59	pF
From sensor to PAD	Metal width	5	μm
	Metal length	5500	μm
	Line R	2.2	kΩ
	Line C	2.901	fF
FPC cable	Unit R per mm	0.035	Ω
	Line R	2.17	Ω
	Unit C per mm	7.147	fF
	Line C	0.443	pF
On PCB	Line R	121	Ω
	Line C	2.45	pF
Total R		6.323	kΩ
Total C		4.485	pF

Table S2. Parasitic resistance (R) and capacitance (C) values calculated from the fingerprint sensing system in order to confirm the operation speed of system. The remaining parasitic capacitances result from a metal line from the sensor to the pad, the flexible printed circuit (FPC) cable, and the printed circuit board (PCB) except for the parasitic capacitance of fingerprint sensor. The parasitic resistance is composed of the metal line in the sensor, the metal line from the sensor to the pad, the metal line in the FPC cable, and the metal line on the PCB.
